# Current developments in elastic and acoustic metamaterials science

**DOI:** 10.1098/rsta.2024.0038

**Published:** 2024-09-23

**Authors:** Giuseppe Failla, Alessandro Marzani, Antonio Palermo, Andrea Francesco Russillo, Daniel Colquitt

**Affiliations:** ^1^ Department of Civil, Energy, Environmental and Materials Engineering (DICEAM), University of Reggio Calabria, Via Zehender, Reggio Calabria 89124, Italy; ^2^ Department of Civil, Chemical, Environmental and Materials Engineering (DICAM), University of Bologna, Viale del Risorgimento 2, Bologna 40136, Italy; ^3^ Department of Mathematical Sciences, University of Liverpool, Liverpool L69 7ZL, UK

**Keywords:** metamaterial, elastodynamics, acoustics, design, computational modelling, wave control

## Abstract

The concept of metamaterial recently emerged as a new frontier of scientific research, encompassing physics, materials science and engineering. In a broad sense, a metamaterial indicates an engineered material with exotic properties not found in nature, obtained by appropriate architecture either at macro-scale or at micro-/nano-scales. The architecture of metamaterials can be tailored to open unforeseen opportunities for mechanical and acoustic applications, as demonstrated by an impressive and increasing number of studies. Building on this knowledge, this theme issue aims to gather cutting-edge theoretical, computational and experimental studies on elastic and acoustic metamaterials, with the purpose of offering a wide perspective on recent achievements and future challenges.

This article is part of the theme issue, ‘Current developments in elastic and acoustic metamaterials science (Part 2)’.

## 1. Introduction

This is Part 2 of the theme issue ‘Current developments in elastic and acoustic metamaterials science’, which includes contributions on design methodologies and typologies, modelling and analysis and engineering applications of elastic and acoustic metamaterials. For a general overview of these topics, readers are referred to the introduction article to Part 1.

Here follows a description of the works included in Part 2 with a brief outline of the most recent pertinent literature.

## 2. Design methodologies and typologies

In recent years, topological physics appeared as a novel research area and a valuable source of innovative design methodologies for materials and devices across various engineering applications. Essentially, topological physics seeks to uncover how the geometry and topology of the environment, where physical processes occur, influence those processes themselves [1].

Within the domains of topological elasticity and acoustics, current research aims to support the development of novel engineered materials like elastic and acoustic topological metamaterials. The main purpose is to leverage topological principles to robustly manipulate elastic and sound waves and achieve protection against backscattering caused by defects in waveguides. The applications of topological protection in elastic and acoustic wave contexts are numerous, and interested readers are encouraged to consult recent reviews for a comprehensive overview of these topological waveguides, such as the works in Miniaci & Pal and Xue *et al.* [2,3] and others. A fundamental role in understanding the mathematical foundations and design principles of elastic and acoustic topological metamaterials is played by the concept of geometric phase. The geometric phase refers to the phase difference acquired by the response of a system over the course of a cycle when the system is subjected to cyclic adiabatic variations of its parameters; in this context, the term adiabatic is used in its quantum mechanical sense indicating a slowly varying quantity. In the theme issue, Kumar *et al.* [4] revisit the concept of geometric phase in elastic waves and its applications for designing elastic metamaterials. The occurrence of a geometric phase in the response of quasi-one-dimensional elastic waveguides is shown through theoretical explanations and practical examples as, e.g. an isotropic waveguide with adiabatically varying triangular cross-section and a helical waveguide created by sweeping a circular cross-section through a generating path in space. The authors demonstrate that the geometric phase depends solely on the path traced in the parameters space (rather than on the rate of parameter change) and that the geometric phase can be classified as either topological or non-topological, with only the former proving robust against small perturbations to the design of the waveguide. Moreover, concepts from differential geometry, such as fibre bundles and parallel transport, are employed to provide a theoretical foundation and interpretation of the geometric phase, with applications to the design of elastic topological metamaterials. Interestingly, the authors conclude that elastic topological metamaterials can be described as fibre bundles classified according to topological invariants and demonstrate, on this basis, that the interface of elastic topological metamaterials with differing topological invariants can support localized edge modes topologically protected against perturbations to the properties of the waveguide.

Besides topology, recent design methodologies for elastic and acoustic metamaterials leverage non-local couplings, specifically beyond-nearest-neighbour (BNN) interactions, to control their dynamic properties. Interest in BNN interactions dates back to Brillouin, who discussed dispersion relations in periodic lattices with BNN couplings [5]. Metamaterials designed with BNN interactions exhibit ‘roton-like’ dispersion curves with local minima indicating group velocity inversion, a concept akin to Landau’s rotons in superfluid helium [6]. Experimental observations confirmed these features in various elastic and acoustic metamaterials [7,8]. Recently, inverse-design techniques for mass-spring chains with desired phononic dispersion curves utilized non-local interactions [9]. In the theme issue, Guarracino *et al.* [10] focus on one-dimensional monoatomic non-local lattices made of masses (atoms) equally distanced from each other and mutually connected by linearly elastic springs. Under the assumption of periodicity in the system, the authors establish a design framework for the construction of BNN lattices with varying degrees of non-locality (*P*), which involves a constraint on the constant total mass of the springs branching off from each single atom, regardless of the number of BNN pairings, and a general exponential law to describe spring stiffness scaling with distance, characterized by a parameter α . Analytical derivations of dispersion relations, including computations of group velocities, are provided as functions of α and *P*. Qualitative results and useful inequalities are complemented by outcomes from numerical simulations and two-dimensional fast-Fourier transforms on representative examples, illustrating key dynamic characteristics of finite-length lattices across different α and *P* values. In particular, the study explores the implications of enforcing additional BNN couplings in a scenario where the amount of material is limited and an order-of-magnitude mass ratio is enforced between springs and atoms. The proposed design framework is also of interest for a rational comparison between local and non-local lattices, on the basis of different distributions and arrangements of the same amount of raw material. The study includes a discussion of examples of non-local connectivity in realistic materials and potential experimental validations of the proposed concepts.

## 3. Modelling and analysis

Elastic metamaterials with chiral microstructure, i.e. not invariant concerning reflection and equipped with internal rotational units, gained considerable attention from researchers as effective media with remarkable auxetic properties. The seminal paper in Prall and Lakes [11] proposed an auxetic triangular lattice composed of circular rings each connected to neighbouring rings by straight ligaments tangent to the rings, where the rotation of the rings induced by stress fields results in auxetic behaviour with Poisson’s ratio almost equal to −1. Building on this work, several studies developed two-dimensional chiral lattice metamaterials and three-dimensional generalizations to attain anisotropic and isotropic auxetic properties [12,13]. The analysis of chiral microstructures is challenging and requires specific computational methods, e.g. homogenization techniques. In the theme issue, Bacigalupo *et al*. [14] present a stratified metamaterial built by stacking two-dimensional lattice layers with alternate hexachiral topology, each layer consisting in a periodic assembly of solid circular discs connected by elastic slender prismatic ligaments; moreover, the layers are interconnected through elastic pins passing through the centres of aligned discs. The authors adopt a micropolar continuum model for each layer and describe at the continuum level the interaction between layers. The interaction occurs via the pins and depends on the relative displacement between adjacent discs, including relative rotation around the pins. As a result, the response of the stratified metamaterial is obtained via a multifield non-local model involving average and difference of displacement and rotational fields of the discs. Closed-form expressions are obtained for the overall micropolar and standard (Cauchy) constitutive tensors. The equivalent generalized micropolar model is validated against discrete Lagrangian solutions of two representative elasto–static benchmark problems. Interestingly, results show that, by allowing relative rotation between adjacent discs, strong isotropic and anisotropic auxeticity can be achieved even at moderately small chirality angles.

Computational homogenization methods are of particular interest among the many methods devised for analyzing locally resonant elastic and acoustic metamaterials. In the theme issue, Liupekevicius *et al*. [15] develop a computational homogenization method in a pressure-based framework, especially suitable for metamaterials relying on fluid resonance as, e.g. labyrinthine acoustic metamaterials [16–19]. The method delivers an equivalent macro-scale continuum with non-standard constitutive relations retrieved from the microstructural unit cell response. Specifically, the micro-scale dynamics are represented in a reduced space by superposing long wavelength and local resonance responses, and an extended version of the Hill–Mandel principle is applied to formulate a macroscopic homogenized enriched continuum with additional enriching variables describing the microscale dynamics in a subwavelength regime. The method is applicable in both time and frequency domains, without the additional enriching variables in the frequency domain. For illustration purposes, the authors study a two-dimensional acoustic metamaterial, the unit cell of which contains a rigid solid labyrinthine-like structure immersed in a fluid, where the coiled space region triggers the fluid resonance inside the rigid structure in a subwavelength regime relative to the non-coiled region. The method proves capable of capturing exotic macroscopic responses typically found in acoustic metamaterials, e.g. negative effective properties and Willis coupling, lending itself to further potential developments, as discussed thoroughly in the paper [15].

Recently, quasi-periodic locally resonant elastic metamaterials became a subject of active research in the field [20–22]. Typically, quasi-periodicity is obtained by cyclically modulating geometrical parameters as mutual distance between resonators [23,24], height of resonators [25] or, alternatively, mechanical parameters such as stiffness [26]. In addition to classical local resonance band gaps, quasi-periodic locally resonant metamaterials can have topological band gaps, i.e. frequency bands hosting edge-localized modes in corresponding metamaterial finite structures. These edge modes are immune to the presence of defects or imperfections [27] and can be exploited to induce wave localization and attenuation over multiple frequency bands, with applications in vibration isolation and energy harvesting. In the theme issue, Moscatelli *et al*. [28] focus on quasi-periodic metamaterial lattices constituted by bars connected by slender beams acting as resonators; the position of the slender beams is quasi-periodic and cyclically varied according to a specific, quasi-periodic parameter. For wave propagation analysis, the authors develop a discrete equivalent system from which bulk spectrum, trivial and non-trivial topological band gaps can easily be computed. The condition for the appearance of band gaps in the bulk spectrum is established, band gaps are explicitly obtained for several values of the quasi-periodic parameter and validated by comparison with numerical results. Moreover, it is shown that the discrete problem represents other physical realizations of quasi-periodic metamaterials, making the results applicable in other contexts.

For the modelling and analysis of elastic and acoustic metamaterials, it is important to account for uncertainties. Uncertainties may arise from the manufacturing process, making it difficult to define geometrical and mechanical parameters of the resonators in a purely deterministic setting [29,30]. On the other hand, aperiodic design may be intentionally chosen as effective strategy to enhance elastic wave attenuation properties, as noted above. Therefore, methods to assess the effects of variable parameters of the unit cells on the performances of elastic metamaterials are of primary interest. Studies in this respect were conducted by Jensen [31] on one- and two-dimensional periodic mass-spring structures where periodicity is broken by uncertainties in damping and mass within the unit cell, and in ref [32] on plates with beam resonators affected by geometrical uncertainties. Robust-to-uncertainties optimal designs of elastic metamaterials and periodic structures in general were formulated in refs [33–36]. A few recent works focused on locally resonant metamaterials with uncertain parameters described via interval models [37–39]. In the theme issue, the paper by Van Belle *et al*. [40] focuses on elastic locally resonant metamaterial beams and proposes a systematic framework to assess how vibration attenuation in finite beams is affected by uncertainties in resonator parameters as stiffness, mass and position. Modelling the uncertain parameters as interval variables, two non-intrusive uncertainty propagation approaches, i.e. a global search approach and a machine-learning-based uncertainty propagation approach, are developed to calculate upper and lower bounds for three performance metrics of vibration attenuation, i.e. bandwidth, band centre and root-mean-square band averaged response. Important conclusions of the work are that, although variability of all the considered resonator parameters leads to a general trend whereby broadening corresponds to reduced band-averaged attenuation, there is also potential for broadening in combination with improved band-averaged attenuation. This is obtained by an ad hoc design optimization procedure proposed in the paper, along with a robustness assessment procedure. As for computational costs, the machine-learning-based uncertainty propagation approach is found to be a viable alternative to the more exhaustive global search approach, especially for practical cases where the number of uncertain variables is limited.

## 4. Engineering applications

Metamaterial concepts inspired the design of large-scale metamaterial structures with enhanced vibration attenuation properties for several engineering applications [41–45]. Among others, studies explored the potential of systems assembling flexible components and rigid masses [46] that, for example, are relevant to modelling buildings or underwater pipelines. This is the context of the paper by Rosic *et al*. [47] included in the theme issue, which investigates elastic wave propagation in a metastructure conceived as a periodic assembly of Timoshenko beams interconnected by rigid bodies. The unit cell includes a rigid body suspended between two beams with non-aligned longitudinal axes; as a result, transverse and axial displacements are coupled, enriching the dynamics of the cell. A transfer matrix method is developed to calculate the band structure and validated by comparison with results from the finite-element method. Parametric analyses are conducted to demonstrate the effect of the size of the rigid body and beam material properties on size and position of the band gaps. Generalizations of the proposed model of unit cells are discussed throughout the paper.

Motivated by the wide range of large-scale structures that exhibit gyroscopic behaviour, such as wind turbines, a further paper in the theme issue by Madine and Colquitt [48] studies the coupling between transverse, axial and torsional displacements in gyroscopic systems with Euler–Bernoulli beams and thin elastic plates. In particular, a gyroscope is mounted on a structure comprising of two orthogonal Euler–Bernoulli beams. The presence of the gyroscope together with the orthogonal orientation of the two beams results in a dynamic system in which transverse, axial and torsional deformations are fully coupled; this coupling leads to a diverse and complex dynamic response that can be readily tuned. For example, the authors observe what they term ‘dynamic chiral Chladni-like patterns’, which can be controlled using the rate of spin of the gyroscope. The authors develop an analytical model of the system for which the eigenmodes can be found in closed form. The analytical model is complemented by finite-element simulations which both illustrate the dynamic behaviour of the system and validate the analytical solutions. The authors also derive a set of extended chiral-torsional boundary conditions, which allow this complex system to be modelled by a single Euler–Bernoulli beam through the use of a special boundary condition.

As for large-scale applications of metamaterial concepts, the so-called seismic metamaterials have been the subject of very active research in recent years. In a broad sense, the driving principle of seismic metamaterial design is to appropriately modify the properties of the soil surrounding the structure to be protected. This is possible either by soil structuring with periodic cavities, resonant/non-resonant inclusions below the soil surface or, alternatively, by deploying periodic arrangements of external resonant units above the soil surface, attaining seismic wave attenuation via Bragg scattering and/or local resonance phenomena. In the theme issue, Russillo *et al*. [49] proposed an innovative concept of metabarrier for seismic Rayleigh wave attenuation, consisting of a periodic array of cylindrical water tanks acting as resonant units above the soil surface. The theoretical framework involves treating the dynamics of the water tank by a well-established three-dimensional linear, pressure-based model for fluid–structure interaction under earthquake excitation, which accounts for the flexibility of the tank wall. The Bloch–Floquet dispersion analysis demonstrates the presence of relevant band gaps in the low-frequency range of seismic Rayleigh waves, as well as in the higher frequency range typical of Rayleigh waves caused by other ground vibration sources such as road or railway traffic. The band structure is validated by comparison with the frequency-domain analysis of a soil domain with a finite array of water tanks. All calculations are implemented in Comsol Multiphysics. From a design point of view, the appealing feature of the proposed metabarrier is that the water-tank resonant units are readily tunable by changing the water level, thus modifying opening frequencies/widths of the wave attenuation zones.

Finally, moving from the large scale to the small scale, the theme issue deals with the challenging task of designing and fabricating graded index (GRIN) array elastic metamaterials that can be dynamically tuned post-manufacture. Indeed, the possibility of tuning the material’s functionality once the material has been manufactured may represent a significant step in real-world applications of metamaterials. While some progress was made in this regard—for example, through the use of piezoelectric/piezomagnetic materials, exploiting instabilities, pre-stress and so-called ‘soft smart matter’ materials—many such approaches are difficult to implement in practice. In the theme issue, Dal Poggetto *et al.* [50] present a relatively simple mechanism to tune the mechanical properties, and thereby the dynamic behaviour, of a GRIN array elastic metamaterial composed from photo-responsive pillars of variable height (on the millimeter scale). In particular, the pillars are 3D-printed using a commercial resin and then infused with *Disperse Red 1 Methacrylate*, prior to UV curing. The resulting pillars are photo-responsive—their stiffness reduces when illuminated with visible light, resulting in a corresponding shift in resonant frequency. The GRIN array is first modelled numerically using finite elements, before being fabricated, and its performance is then evaluated experimentally. It is demonstrated that the band structure of the GRIN array can be controlled effectively using simple illumination with visible light—the transmittance of elastic waves at the desired frequency changes by approximately 20 dB during illumination. The work provides another promising, and attractively rather simple, avenue for the dynamic control over elastic metamaterials.

## Data Availability

This article has no additional data.
